# Quasi-Bound State in the Continuum of the Intracoupled All-Dielectric Coherent Metasurface

**DOI:** 10.1021/acs.nanolett.5c05162

**Published:** 2025-12-29

**Authors:** Tzu-Hsiang Liu, Hung-Yi Wu, Wen-Hui Sophia Cheng

**Affiliations:** ‡ Department of Materials Science and Technology, 34912 National Cheng Kung University, 701 Tainan, Taiwan; § Academy of Innovative Semiconductor and Sustainable Manufacturing, 34912 National Cheng Kung University, 701 Tainan, Taiwan; ⊥ Center for Quantum Frontiers of Research and Technology (QFort), 34912 National Cheng Kung University, 701 Tainan, Taiwan; ∥ Center for Resilience and Intelligence on Sustainable Energy Research (RiSER), 34912 National Cheng Kung University, 701 Tainan, Taiwan

**Keywords:** metasurface, dielectric, q-BIC, *Q* factor, photoluminescence enhancement

## Abstract

Metasurfaces have transformed the landscape of nanophotonics by enabling the precise control of light–matter interactions across a wide spectral range. Recently, dielectric metasurfaces have garnered significant attention due to their potential for low nonradiative loss. In this study, we introduce a novel metasurface design that employs biradial circular resonators to induce quasi-bound states in the continuum (q-BIC) through minimal symmetry breaking, demonstrating resonance with a *Q* factor of 650. It is predominantly driven by the interference of the electric dipole and the magnetic quadrupole. This approach addresses key limitations by simplifying fabrication and enhancing robustness while delivering high tunability, strong photonic confinement, and a 4-fold photoluminescence enhancement experimentally. The proposed strategy not only provides a new pathway for emission enhancement but also underscores the broader utility of q-BIC metasurfaces in light–matter interaction. Through a detailed analysis of the symmetry, resonance tuning, and field distribution, we elucidate the fundamental physics underlying our design.

## Graphical abstract

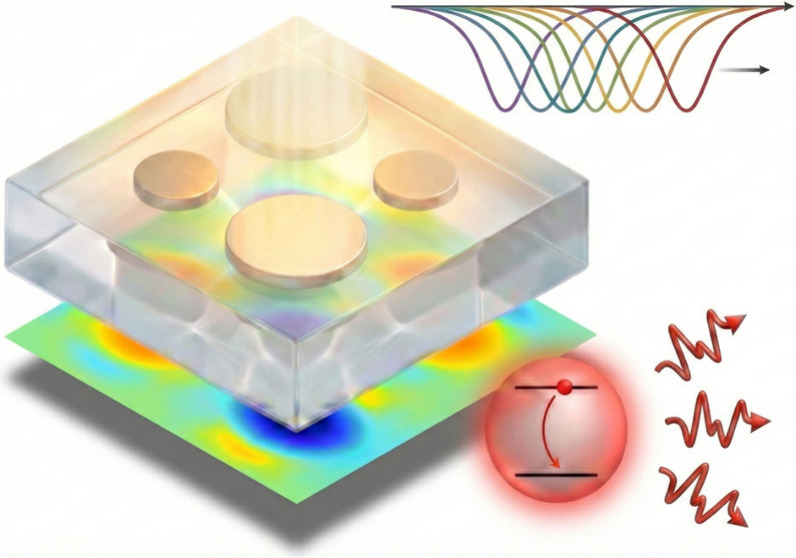

Metasurfaces have emerged as a transformative platform in nanophotonics, offering unprecedented control over all characteristics of light. Their versatility has enabled the development of compact optical components across a broad spectral range.^1–4^ One of the critical advantages of metasurfaces lies in their ultrathin profile and design flexibility, making them ideal candidates for integrated photonic systems in sensing,^5,6^ imaging,^7^ and optical communication.^8,9^ Traditionally, plasmonic metasurfaces composed of noble metals have been widely investigated due to their ability to confine light to volumes that are deeply subwavelength in scale via surface plasmon resonances. However, the severe intrinsic ohmic losses significantly degrade their efficiency, limiting their practicality in applications requiring high-*Q* resonances and minimal energy dissipation.^10,11^

To overcome these limitations, recent research has shifted toward all-dielectric metasurfaces composed of high-refractive-index materials. These structures support Mie-type and Fano resonances with substantially lower intrinsic absorption, enabling improved efficiency and performance.^11–13^ However, dielectric metasurfaces still experience radiative losses, from energy leakage into the far field and scattering losses, due to fabrication imperfections. These become critical when targeting narrow line widths or strong field confinement. Thus, further suppression of these losses is essential to fully realizing the potential of metasurfaces.

A powerful solution to this problem lies in the concept of bound states in the continuum (BIC). BIC has gained substantial traction in photonics due to its ability to trap light without radiative loss despite being energetically degenerate with continuum modes.^14^ In metasurfaces, BIC can be realized through symmetry protection or destructive interference among radiative channels.^15–17^ Ideally, such states are perfectly confined, yielding theoretically infinite *Q* factors. However, because ideal BICs are decoupled from the far field, they are unobservable in practice.

By introducing controlled symmetry breaking, the ideal BIC can be transformed into a quasi-BIC (q-BIC), a leaky resonant state with finite but an ultrahigh *Q* factor that couples weakly to the radiation continuum. This transformation opens the door to practical applications because q-BIC retains the desirable features of strong field localization and sharp resonances while becoming experimentally accessible.^18,19^ Recent designs have demonstrated advanced functionalities such as ultrafast modulation^20,21^ and spin-selective dual q-BIC.^22^ Hyperspectral imaging, near-perfect light absorption, and sensing applications leveraging q-BIC have also been explored.^23–25^

Numerous approaches have been proposed to achieve q-BIC, including asymmetric nanobar arrays, elliptical perturbations, displaced dimers, and tilted nanostructures.^26–30^ However, these methods often involve complex geometries, stringent fabrication tolerances, or reliance on nanoscale gaps, which can hinder large-area fabrication and robustness. In this work, we present a simple and fabrication-friendly dielectric metasurface design that supports the high-*Q* q-BIC mode using a biradial configuration of circular resonators. The metasurface is composed of a periodic arrangement of supercells, each consisting of four circular dielectric disks with two distinct radii placed diagonally opposite each other, preserving *C*_4_ rotational symmetry. While the geometric structure maintains this high-order symmetry, the use of linearly polarized light breaks the overall excitation symmetry condition, thereby perturbing the ideal BIC and enabling weak coupling to the far field. Compared to previous symmetry-breaking strategies, our design not only maintains fabrication tolerance and modular spectral tunability through geometric parameters but also extends the concept to the near-infrared regime and provides a more detailed analysis of the underlying q-BIC mode.

Among the many applications of q-BIC, we specifically focus on photoluminescence (PL) enhancement, which is of growing interest in fields such as on-chip light sources, bioimaging, and quantum emitters.^20,23,31–33^ While other PL enhancement strategies include plasmonic antennas, photonic crystal slabs, or optical microcavities, each has its limitations.^34,35^ Plasmonic structures suffer from energy losses, while photonic cavities require complex three-dimensional fabrication and often entail strict alignment and coupling constraints.^36^ In contrast, q-BIC metasurfaces offer a planar, low-loss, and design-tunable platform capable of significantly increasing the local density of optical states, thereby enhancing spontaneous emission rates via the Purcell effect.^26,37,38^

Figure 1a illustrates the proposed metasurface unit cell design composed of alternating silicon disks on top of the sapphire substrate. In this model, we adopt a linearly polarized light source along the *x* direction with propagation along the −*z* direction. In consideration of the precise control of the radiative loss of the q-BIC resonance, we propose a parameter called the asymmetry factor (α) to govern the unit cell design. To describe the relationship between α and unit cell parameters, we employed the equations as follows:

$$R_{\mathrm{ave}}=\left(R+r\right)/2;\;R=R_{\mathrm{ave}}\left(1+a/2\right);\;r=R_{\mathrm{ave}}\left(1-a/2\right)$$

where *R* and *r* are the larger and smaller radii of the metasurface and *R*_ave_ is the average radius.

**Figure 1:**
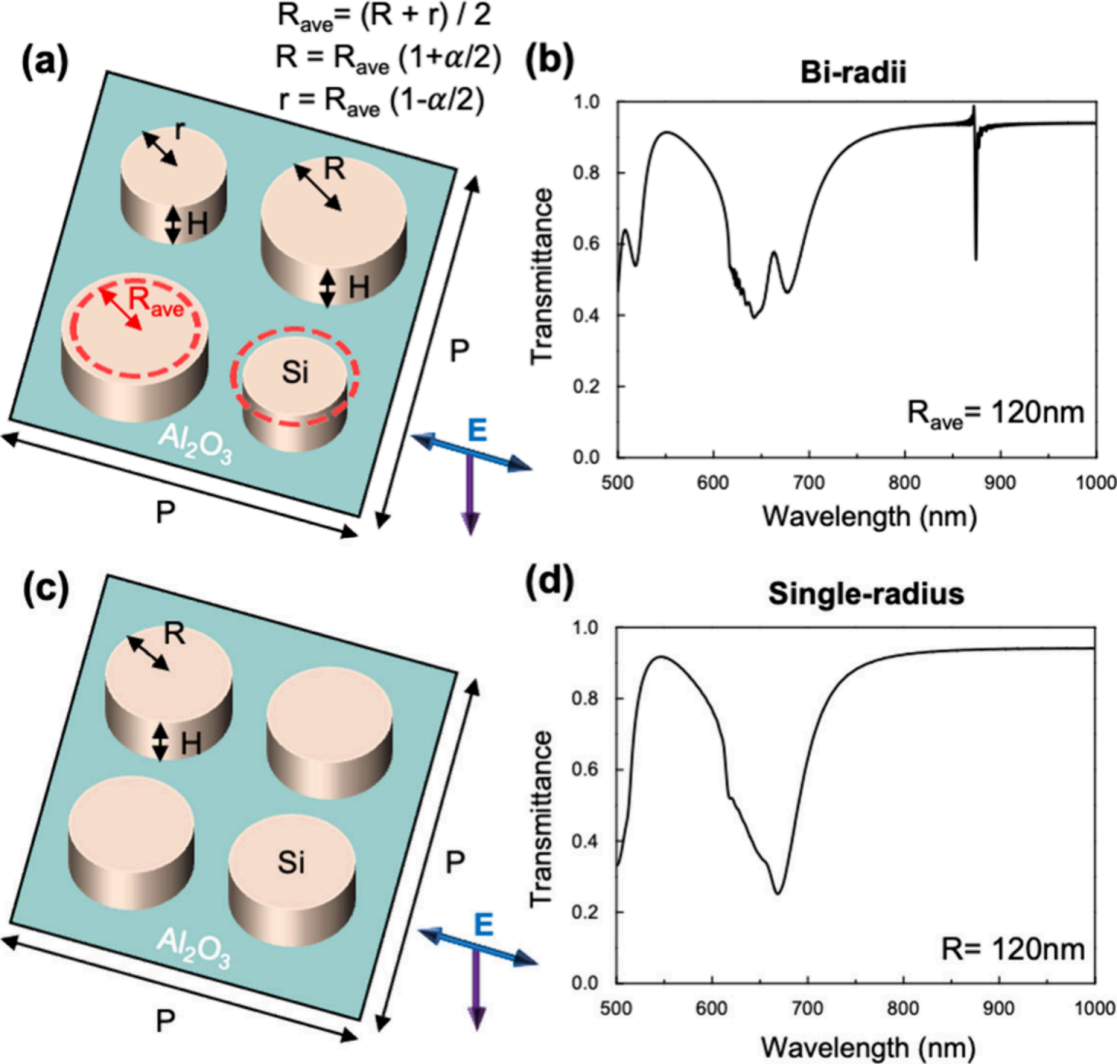
(a) Schematic illustration of the metasurface with an asymmetric design. *R* and *r* are the larger and smaller radii of the metasurface, *R*_ave_ is the average radius, α is the asymmetry factor, the pitch of the unit cell is *P*, and the height is *H*. (b) Simulated transmittance spectrum corresponding to the biradii structure with *R*_ave_ = 120 nm, α = 0.1, *R* = 126 nm, *r* = 114 nm, *P* = 700 nm, and *H* = 100 nm. (c) Schematic illustration of the symmetry metasurface. *R* is the radius. (d) Simulated transmittance spectrum corresponding to the single-radius structure with *R* = 120 nm, *P* = 700 nm, and *H* = 100 nm. The metasurface is illuminated by the *x*-polarized plane waves propagating along the −*z* axis, as indicated by the blue and purple arrows, respectively.

To understand how the biradii metasurface differs from the symmetry cases, we also conduct a simulation on the metasurface consisting only of a uniform Si disk on the substrate, as shown in Figure 1c. To investigate the characteristics of these metasurfaces, we simulate the corresponding transmittance spectra of the metasurfaces under periodic boundary conditions, as depicted in Figure 1b,d. The results reveal that both designs exhibit broadband resonances in the visible range with overall similar spectral trends, although subtle differences arise due to their structural distinctions. Evidently, with the asymmetry introduced into the structure, we achieve the q-BIC state at 874.8 nm in the near-infrared (NIR) wavelength range.

To gain a deeper understanding of the q-BIC-induced polarization, we first analyze the field profile. The positions of the field monitor at *xz* and *xy* planes are illustrated in Figure 2a. Figure 2b indicates that there is a strong electric-field enhancement at the bottom corner of both silicon disks. Also, a strong magnetic field is exhibited in the middle of the structure shown in Figure 2c. These strongly localized field enhancements are solid evidence that a resonance exists and energy is preserved. Furthermore, we resolve the electromagnetic fields in diverse directions for a complementary understanding of the resonance, as shown in Figures S1 and S2. The results point out that the strong electric field in the *E*_*x*_ and *E*_*z*_ components substantially causes the electric-field enhancement, and the strong magnetic-field enhancement is contributed by the magnetic field in the *H*_*y*_ component. The clear quadrupolar-like field distribution, as shown in Figure 2d, supports the formation of a magnetic quadrupole.

**Figure 2:**
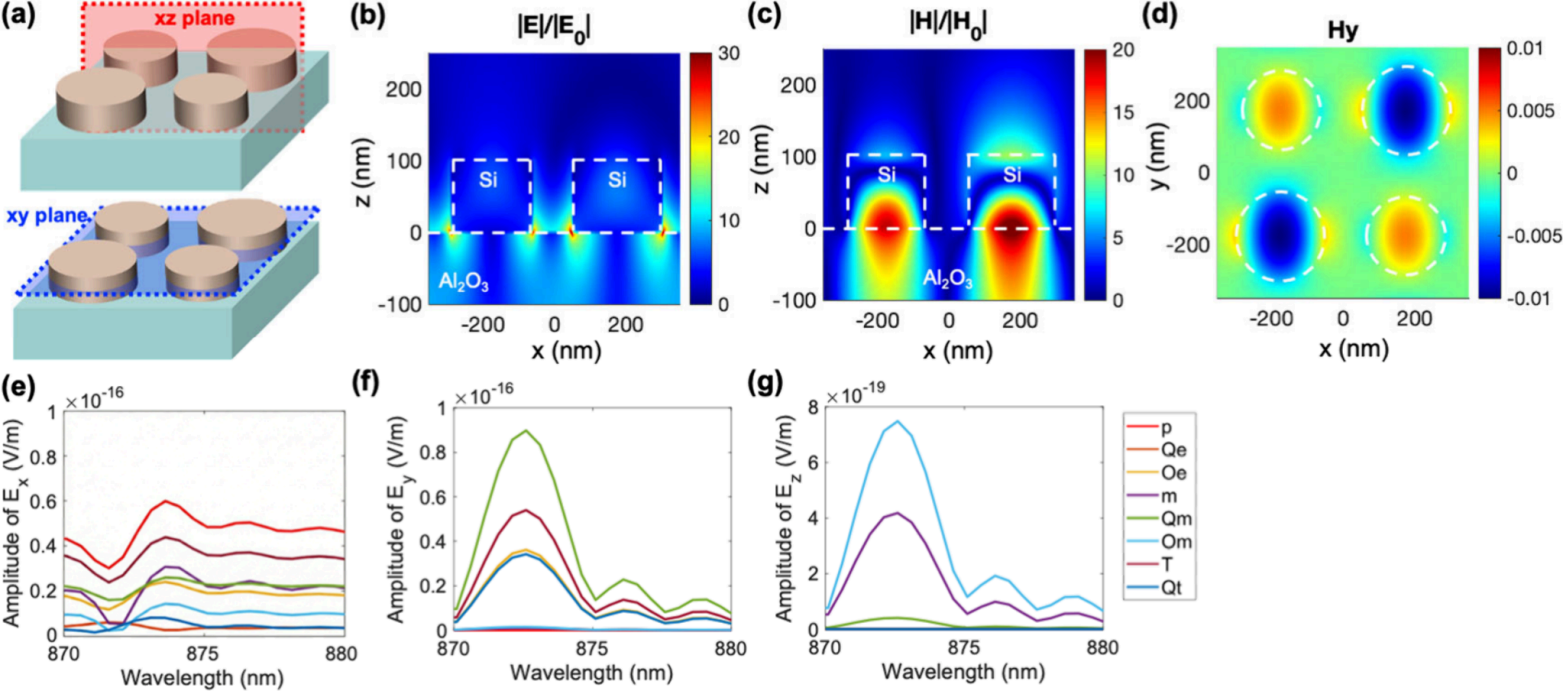
(a) Schematics showing the field monitor at the *xz* and *xy* planes. (b) Normalized electric-field distribution at the *xz* cross section of the metasurface. (c) Normalized magnetic-field distribution at the *xz* cross section of the metasurface. (d) Resolved magnetic fields correspond to *y* components at the *xy* cross section of the metasurface. (e–g) Multipole decomposition analysis of the resonance of *E*_*x*_, *E*_*y*_, and *E*_*z*_ components. The contributions of the electric dipole (p), electric quadrupole (Qe), electric octupole (Oe), magnetic dipole (m), magnetic quadrupole (Qm), magnetic octupole (Om), toroidal dipole (T), and toroidal quadrupole (Qt) are indicated accordingly. For the biradii structure, *R*_ave_ = 120 nm, α = 0.1, *R* = 126 nm, *r* = 114 nm, *P* = 700 nm, and *H* = 100 nm.

Moreover, we conducted a multipolar decomposition analysis to delve into the origin of the resonance, as depicted in Figure 2e–g. The results reveal that the resonance is primarily induced by the electric dipole in the *E*_*x*_ component and the magnetic quadrupole in the *E*_*y*_ component. On the basis of the field profiles and multipolar decomposition, we conclude that the q-BIC resonance in the biradii metasurface is predominantly driven by the interference of the electric dipole and the magnetic quadrupole.

For comparison, we investigate the single-radius metasurface at 874.8 nm, and it turns out to show a weak field enhancement in Figure S3. Notably, the dominant multipole is only the electric dipole in the *E*_*x*_ component, with the absence of the magnetic quadrupole, which indicates no localized states, as shown in Figure S4. These results emphasize the crucial role of the magnetic quadrupole in creating the q-BIC resonance. Since the coupling environment influences the resonance condition, we explore the resonance conditions under diverse surroundings, as shown in Figure S5. We notice that there is an additional resonance at a shorter wavelength range when the surroundings tend to be uniform, which means the surrounding refractive index is close to 1.75 (index of a sapphire substrate). We conduct multipole decomposition on the q-BIC resonance in a uniform (*n* = 1.75) environment to understand the resonant condition. Upon considering all components, we observe that the dominant multipoles for the emerging resonance are an electric dipole in the *E*_*x*_ component and the toroidal quadrupole in the *E*_*y*_ component.

By applying the asymmetry factor α, we are capable of manipulating the radiative loss of the resonance, which allows us to suppress the total energy loss. As can be observed in Figure 3a, the presented transmittance spectra of α values ranging from 0.05 to 0.5 clearly exhibit variation of the resonance condition. We observe that when α becomes larger, the full width at half-maximum (fwhm) of the resonance also becomes wider, which means the energy loss becomes larger. This is because, with increasing α, the radiative decay rate of the state that describes the interaction between the near field and the far field becomes larger. As a result, larger energy is emitted to the far field. To know exactly how α influences the q-BIC resonance, we quantitatively measure the fwhm of the q-BIC resonance by fitting the spectrum with Lorentz’s relationship and calculating the *Q* factor according to the following equation:

$$Q=\frac{\lambda_{r}}{△\lambda }$$

where λ_r_ is the resonant wavelength and Δλ is the fwhm of the resonance. As illustrated in Figure 3d, the *Q* factor increases with the decreasing asymmetry factor, and the trend follows the inverse quadratic relationship, which is a manifestation of q-BIC.^26^ Notably, with an asymmetry factor equal to 0.05, a *Q* factor of 650 is attained.

**Figure 3:**
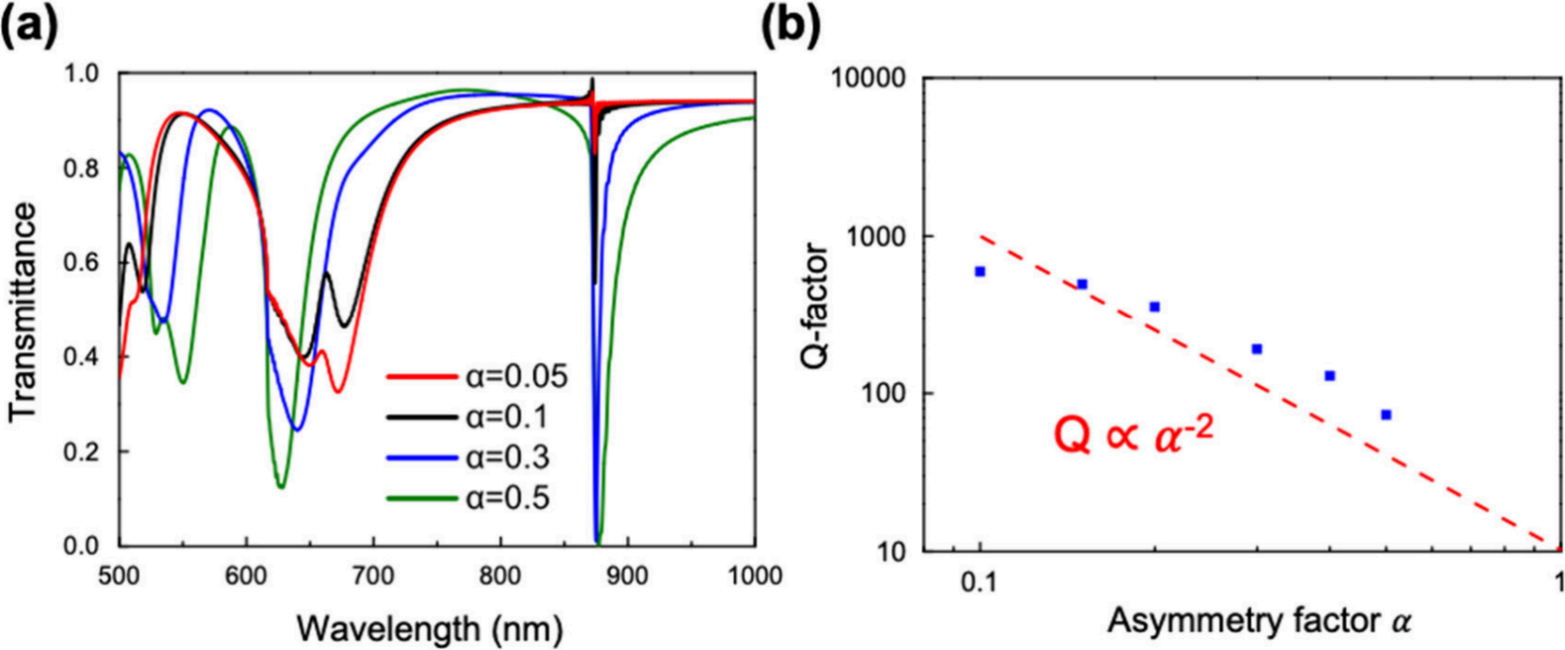
(a) Transmittance spectrum of the metasurface with varied asymmetry factors α (*R*_ave_ = 120 nm, *P* = 700 nm, and *H* = 100 nm). (b) Relationship between the asymmetry factor α and the calculated *Q* factor. The inverse quadratic relationship is indicated by the red dashed line.

Because the q-BIC resonance is susceptible to the coupling condition, as previous results revealed, we additionally explored the impact of the periodicity arrangement and structure size, as presented in Figure 4. It has been observed that altering the pitch of metasurfaces notably affects their optical response. To investigate this phenomenon, we conduct the transmittance mapping and analysis of a biradii metasurface with varying *P*, as depicted in Figure 4a–c. The diagram suggests that the q-BIC resonances with increased unit cell sizes present a redshift. The implication behind this shift is that the lower density of the nanodisks in the metasurface leads to weaker interactions between the incident light and nanoparticles, resulting in resonance with a longer wavelength.

**Figure 4:**
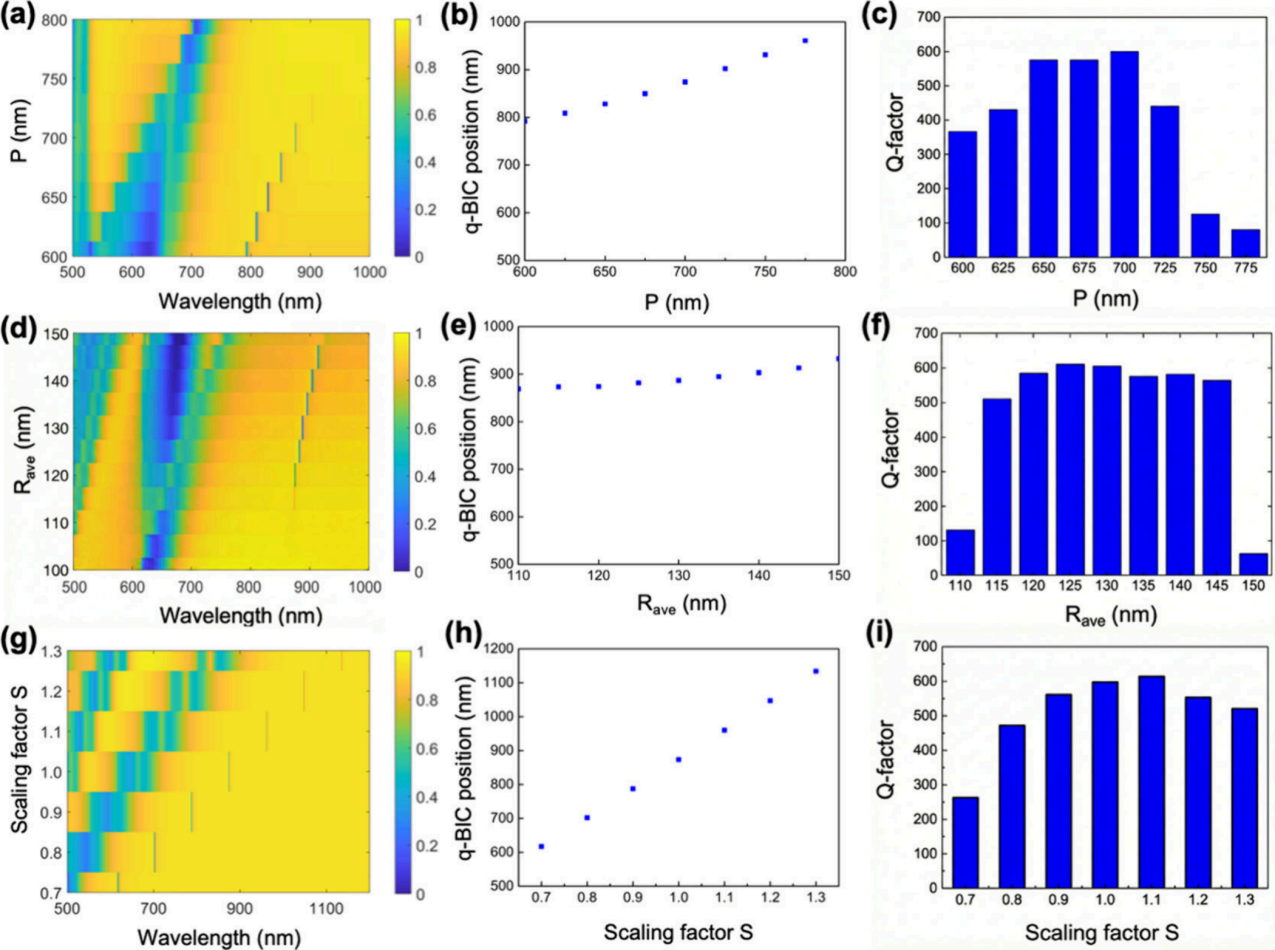
(a) Transmittance mapping of the biradii metasurface with varied *P* (*R*_ave_ = 120 nm, *H* = 100 nm, α = 0.1, *R* = 126 nm, and *r* = 114 nm). (b) q-BIC resonance position corresponding to part a. (c) *Q* factor of the q-BIC resonances corresponding to part a. (d) Transmittance mapping of the biradii metasurface with varied *R*_ave_ (*P* = 700 nm, *H* = 100 nm, α = 0.1, *R* = 126 nm, and *r* = 114 nm). (e) q-BIC resonance position corresponding to part d. (f) *Q* factor of the q-BIC resonances corresponding to part d. (g) Transmittance mapping of the metasurface with varied scaling factor *S*. (h) q-BIC resonance position corresponding to part g. (i) *Q* factor of the q-BIC metasurface corresponding to part i.

A redshift of approximately 200 nm is observed when the pitch is altered from 600 to 775 nm. However, it is shown that the resonance becomes increasingly faint as the pitch expands beyond 700 nm and completely disappears when the pitch reaches 800 nm. Apart from the presence of the resonance, the corresponding *Q* factor was computed to evaluate the resonance condition. The high *Q* factor can be realized only within a limited pitch range. We also discuss the dependency of the nanodisk *R*_ave_ on q-BIC, as shown in Figure 4d–f. The results reveal that the q-BIC resonance is absent at a metasurface radius of less than 110 nm and is maintained over a wide range of radii. However, if the metasurface radius becomes too large, then the resonance becomes broader and tends to disappear. Also, we calculate the corresponding *Q* factor and notice that it can be kept at a high level from 115 to 145 nm.

For the q-BIC resonance, it is crucial to know the criteria for the maintenance of the high *Q* factor. We exploit the multipole decomposition analysis with different *P*, as shown in Figure S6. It is clearly observed that when the pitch is too large to support the considerable magnetic quadrupole (*P* > 700 nm), the corresponding *Q* factor drops rapidly. We attribute this phenomenon to the incomplete cancellation of the electric dipole and magnetic quadrupole. Because all of the evidence indicates that the presence of the magnetic quadrupole plays a crucial role, we decompose the influence of *P* to delve further by fixing the pitch along one direction and changing another, as presented in Figure S7. It turns out that the q-BIC resonance shows the equivalent dependence of pitches along both directions, which is because the magnetic quadrupole is induced by the near-field coupling of the states along both the *x* and *y* directions.

To elucidate the situation, we analyzed the metasurface with a unit cell consisting of only one symmetry plane for a complementary understanding, as shown in Figure S8. After studying the metasurface with a diverse average radius and asymmetry factors, we are convinced that the magnetic quadrupole induced by the asymmetry arrangement in both directions is key in inducing the q-BIC resonances. It is noted that the metasurface being analyzed in this section also follows the designed principle mentioned. Therefore, we conclude that, even with breaking symmetry in the metasurface, it is not always feasible to directly create a new state without considering the composition of the unit cell.

As shown in previous sections, modifying the pitch and radius of the q-BIC structure can alter the interaction between light and matter, thereby causing a shift in the resonance. However, it is impractical to rely on only one parameter to modify the system due to the inherent instability of the resonance *Q* factor and the limited tuning window. Modifying a single parameter without considering others could potentially lead to the destruction of the q-BIC state. Instead, the scaling factor (*S*) was introduced to preserve the q-BIC resonance properties. We can scale all of the geometric parameters (*P*, *H*, *R*_ave_, *R*, and *r*) with *S* while keeping the α constant.

Parts g–i of Figure 4 exhibit transmittance mapping and analysis of the metasurface with scaling factors from 0.8 to 1.3. Note that the standard case (*S* = 1) has a parameter identical to that in Figure 1b. As can be easily observed, we are able to precisely design the resonance position with the proper scaling factor in a large spectral range from visible-to-near-IR regions. Because the original goal of the design is to suppress energy loss, maintaining the *Q* factor is necessary. Calculations show that the high *Q* factor can be maintained in the spectral range of 700–1150 nm with a scaling factor of 0.8–1.3, indicating a larger working range than single-parameter manipulation. With the parameter introduced, we provide flexibility while keeping the q-BIC resonance.

The subtle symmetry breaking in our design modulates the interference between the resonant modes, enabling the emergence of a q-BIC with tunable leakage rates. Notably, this method avoids the use of fragile nanogaps or intricate asymmetries, which are often difficult to realize uniformly over large areas. The resulting q-BIC exhibits strong field confinement and narrow line widths, providing a promising platform for light–matter interaction enhancement.

To validate the results obtained from the simulation of the q-BIC biradii metasurface, we fabricate a set of metasurfaces with diverse average radii from 106 to 150 nm, all covering a total area of 100 × 100 μm^2^. We adopt a top-down process with bilayer e-beam resist lithography and deposit a chromium layer as a hard mask. After the dry etching and mask removal process, we acquire the metasurface with silicon disks on a sapphire substrate. For a detailed fabrication process, refer to the experimental part.

Parts a–c of Figure 5 are the SEM images of the metasurface with different *R*_ave_. To understand the optical behavior of the metasurface, we conducted transmission measurements, as depicted in Figure 5d. The experimental setup is presented in Figure S9. For the case with a *R*_ave_ of 132 nm, a q-BIC resonance with a *Q* factor of 120 is successfully induced. Also, we can see q-BIC resonances for diverse *R*_ave_. To confirm the occurrence of the induced q-BIC resonance, we fabricated a metasurface with a single radius of 130 nm, maintaining the same array size. The results are shown in Figure S10.

**Figure 5:**
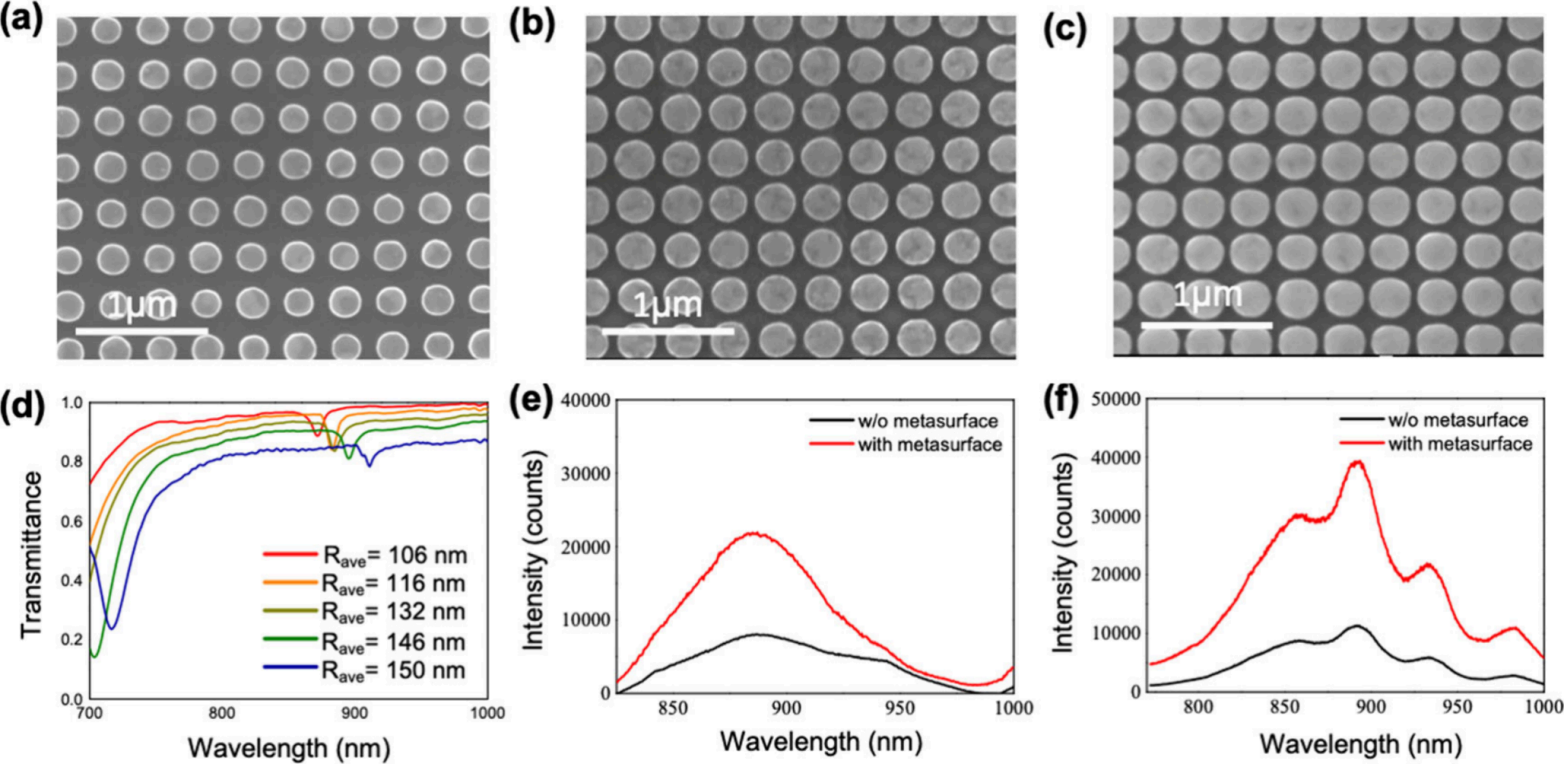
(a–c) SEM images of the biradii metasurface with *R*_ave_ of 106, 132, and 150 nm, respectively. (d) Transmittance spectra of the biradii metasurface with different *R*_ave_. (e) PL spectra of CF@870 dye emitter with and without a biradii metasurface. (f) PL spectra of PbS quantum dots with and without a biradii metasurface.

In the transmittance spectrum of a single radius metasurface, we observe no q-BIC resonance, as predicted by the simulation. Therefore, we are convinced that a resonance mode made accessible through asymmetry-induced radiation coupling can be realized with our biradii design. We notice discrepancies between the simulation and experiment upon comparison of Figure 5d with Figure 1b. The *Q* factor of the resonance is lower than the simulated result. We attribute this to the nonideal fabrication process of the metasurface, causing an averaging effect of the spectrum and broadening the line width of the q-BIC resonance. For the investigation of the impact of *R*_ave_ on the metasurface optical properties, we observe a redshift of the q-BIC as *R*_ave_ increases, which agrees with the predictions from our simulations. This finding supports our simple design principle in achieving robust q-BIC metasurfaces.

To demonstrate the benefit of the metasurface with induced q-BIC, we conducted PL measurements. In this study, we choose CF@870 dye as the emitter whose absorbance peak is at 876 nm, as shown in Figure S11. We adopted a microscope system to collect the PL signals, as shown in Figure S12. A bandpass filter allows excitation of dye at 725–825 nm, and the PL emission spectra are collected above 825 nm. We select the metasurface with an *R*_ave_ of 132 nm to characterize the PL enhancement from the q-BIC resonance. Notably, we can observe the signal at around 885 nm when measuring the PL signal without the metasurface, as is evident in Figure 5e. After introducing a metasurface, we obtain a 4-fold PL enhancement with the help of the q-BIC resonance. This is because the q-BIC resonance increased the density of the optical states, corresponding to Purcell enhancement.

To check the robustness of our q-BIC resonance, we conducted another set of measurements with different types of emitters, the PbS quantum dots. The absorbance of the quantum dots increases gradually at shorter wavelengths with a clear shoulder peak around 783 nm (Figure S11). The measurement setup is presented in Figure S13. A bandpass filter of 530–550 nm is chosen instead to allow complete isolation of absorption from emission, and the PL emission spectra are collected above 775 nm. As can be seen in Figure 5f, we can observe a trend similar to that of the emission from the dye. The observed spectral features reflect slight heterogeneity in the quantum dot size or surface states, leading to broadened and multimodal emission across the NIR range. The PL intensity of the quantum dot on the metasurface is 4-fold larger than the case without the metasurface, which again verifies the ability of the q-BIC resonance to enhance the PL signals. It is worth noting that, due to ultrafast intraband relaxation and ensemble averaging of the dye and quantum dot, this gain propagates across the whole emission band. The PL intensity can be further improved by reducing nonradiative decay through passivation or hybrid materials integration. On the basis of these results, we have successfully demonstrated the capability of the fabricated metasurface with q-BIC resonance to enhance the spontaneous emission.

In the scope of this work, we provide a complementary understanding of the q-BIC metasurface including the origin of the resonance, resonance position manipulation, and the quality preservation of the resonance by simulation design and experimental verification with application. We observed that we could induce an additional resonance with the asymmetric geometry of the silicon disk. The formation of the state depends on the modes being induced and the interference between them. Our findings indicate that the resonance position and the quality are sensitive to the surroundings and geometric design. To preserve the *Q* factor of the resonance, we employed the scaling factor to maintain a considerable value in the spectral range from 700 to 1100 nm.

Moreover, we experimentally demonstrate that the metasurface with a proper design exhibits an extra resonance with a *Q* factor of 120. Besides, we also verified that the metasurface with diverse average radii will have varying resonance positions, as predicted. To realize the applicability of the q-BIC metasurface, we conducted PL experiments as a proof of concept. With the metasurface, we can enhance the PL signal by 4-fold. Overall, this study has provided valuable insights into the q-BIC metasurface and its resonance properties. The versatile design would benefit photonic applications. For example, by precisely tuning the radiation loss of the resonance through the q-BIC concept to match the nonradiative loss of the material, superabsorption can be realized and employed in the energy field with proper material selection and structural design.

## Supplementary Material



## Data Availability

The data that support the findings of this study are available from the authors on reasonable request.

## Notes

Method, field profile analysis of the metasurface, multipole decomposition of the single radius metasurface, background index dependence, multipole decomposition analysis with different pitches, transmittance mapping of the biradii metasurface, schematic illustration and transmittance mapping of the half-biradii metasurface unit cell, multipole decomposition of the half-biradii metasurface with varied asymmetry factor (α), SEM images and transmittance spectra of the fabricated single radius metasurface, optical setup for transmission and PL measurement, absorbance spectra of the CF@870 dye and the PbS quantum dots, schematic illustrations of the simulation setup, and schematic illustrations of the fabrication process flow (PDF)
