# Correction to “Quasi-Bound State in the Continuum of the Intracoupled All-Dielectric Coherent Metasurface”

**DOI:** 10.1021/acs.nanolett.6c00966

**Published:** 2026-03-11

**Authors:** Tzu-Hsiang Liu, Hung-Yi Wu, Wen-Hui Sophia Cheng

**Affiliations:** ‡ Department of Materials Science and Engineering, 34912 National Cheng Kung University, 701 Tainan, Taiwan; § Academy of Innovative Semiconductor and Sustainable Manufacturing, 34912 National Cheng Kung University, 701 Tainan, Taiwan; ⊥ Center for Quantum Frontiers of Research and Technology (QFort), 34912 National Cheng Kung University, 701 Tainan, Taiwan; ∥ Center for Resilience and Intelligence on Sustainable Energy Research (RiSER), 34912 National Cheng Kung University, 701 Tainan, Taiwan

In the original paper, there is a typo in the original affiliation of Tzu-Hsiang Liu and Wen-Hui Sophia Cheng.

“Department of Materials Science and **Technology**, National Cheng Kung University, 701 Tainan, Taiwan” should be “Department of Materials Science and **Engineering**, National Cheng Kung University, 701 Tainan, Taiwan”.

