# Supplementary material for: Quasi-Bound State in the Continuum of the Intracoupled All-Dielectric Coherent Metasurface
Source: Nano Lett. 2025 Dec 29;26(1):288–95. doi: 10.1021/acs.nanolett.5c05162 (PMC12810476; doi:10.1021/acs.nanolett.5c05162)
Supplement: Supplementary file 1 [file nl5c05162_si_001.pdf]

Supporting Information

Quasi-Bound State in the Continuum of the Intra-Coupled All-Dielectric Coherent Metasurface

Tzu-Hsiang Liu[1,†], Hung-Yi Wu[2,†], Wen-Hui (Sophia) Cheng[1,2,3,4,]*

[1]Department of Materials Science and Technology, National Cheng Kung University, 701, Tainan, Taiwan

[2]Academy of Innovative Semiconductor and Sustainable Manufacturing, National Cheng Kung University, 701, Tainan, Taiwan

[3]Center for Quantum Frontiers of Research & Technology (QFort), National Cheng Kung University, 701, Tainan, Taiwan

[4]Center for Resilience and Intelligence on Sustainable Energy Research (RiSER), National Cheng Kung University, 701, Tainan, Taiwan

*E-mail: wcheng@gs.ncku.edu.tw

[†] Tzu-Hsiang Liu and Hung-Yi Wu contributed equally to this work.

Methods

Simulations

In the simulation, the Lumerical FDTD software is adopted as our tool for the design of the biradius metasurface including the transmittance spectrum and the electromagnetic field profile. Notably, the multipolar decomposition analysis is also executed in the software, subsequently by the MATLAB data processing. To excite the Q-BIC resonance, we applied the linear-polarized light source along the x direction. The periodic boundary condition is applied along both the x, and y directions, and the

perfect match layer (PML) is set along the z direction (see Figure S13). The formulas for calculating each of the multipoles are also provided (see Figure S14).

Multipolar decomposition

The Mie resonance phenomenon, which is observed in the scattering of electromagnetic waves by a dielectric material, is driven by the interference between multipoles. To manipulate this resonance and achieve desired outcomes, it is necessary to have a deep understanding of the various types of multipole moments that contribute to it. For this purpose, researchers have proposed the utilization of a multipole decomposition method for the analysis of scattered electromagnetic waves from nanostructures. The principle behind it is to calculate the displaced current induced by the designed dielectric material and fit it to the mathematical model of diverse multiples with characteristic dipole moment. The mathematical calculation expressions are provided below:

Electric dipole moment (p) : $p = \frac{1}{i\omega}\int J d^3 r,$

Magnetic dipole moment (m): $m = \frac{1}{2c}\int (r \times J) d^3 r,$

Toroidal dipole moment (T): $T = \frac{1}{10c}\int [(r \cdot J)r - 2r^2 J] d^3 r,$

Electric quadrupole moment (Qe): $Qe_{ab} = \frac{1}{i2\omega}\int [r_a J_b + r_b J_a - \frac{2}{3}(r \cdot J)\delta_{ab}] d^3 r,$

Magnetic quadrupole moment (Qm): $Qm_{ab} = \frac{1}{3c}\int [(r \times J)_a r_b + (r \times J)_b r_a] d^3 r,$

Where J is the total volume current density, r is the position vector, and c is the speed of light propagating in a vacuum, ω is the optical angular frequency, and the a and b represent the x,y and z component in the Cartesian coordinate. The scatting power can be presented by the displaced current induced in the dielectric material.

$$I_p = \frac{2\omega^4}{3c^3}|p|^2,$$

$$I_m = \frac{2\omega^4}{3c^3}|m|^2,$$

$$I_T = \frac{2\omega^6}{3c^5}|T|^2,$$

$$I_{Qe} = \frac{\omega^6}{5c^5}\sum_{a,b} |Qe_{ab}|^2,$$

$$I_{Qm} = \frac{\omega^6}{40c^5}\sum_{a,b} |Qm_{ab}|^2$$

Asymmetry Factor α and Q-factor Scaling

In ideal bound states in the continuum, symmetry prevents radiation into free space. However, introducing a small perturbation (e.g., geometric asymmetry) weakly couples the BIC to the radiation continuum, producing a q-BIC, a resonance with a finite but very high Q-factor. The radiation loss rate γ_{rad} of a q-BIC is proportional to the square of the symmetry-breaking parameter α ($\gamma_{rad} \propto \alpha^2$). We define the asymmetry factor α as the relative size difference between the two radii in the biradial metasurface:

$\alpha = (R - r) / R_{ave} = 2(R - r) / (R + r)$

When R = r, α = 0 and the structure is fully symmetric, supporting a symmetry-protected BIC. As α increases, symmetry is broken and the mode leaks into the far field, forming a q-BIC. From temporal coupled-mode theory, for a resonance weakly coupled to radiation due to small symmetry breaking:

$a(t) = a_0 e^{(i\omega_0 t - \gamma t)}$

where $\gamma = \gamma_{rad} + \gamma_{nr}$ is the total decay rate, dominated by radiative decay. Since the Q-factor is inversely proportional to the total loss rate (mainly dominated by radiative loss in our low-loss dielectric system), we obtain:

$Q = \omega_0 / (2\gamma) \approx \omega_0 / (2\gamma_{rad}) \propto \alpha^{-2}$

This scaling has been analytically derived and numerically verified in the literature. As shown in Figure 3b of the main manuscript, we numerically extracted Q-factors for

different values of α and confirmed the expected scaling. The result follows a $Q \propto \alpha^{-2}$ trend, validating the q-BIC origin of the resonance.

Fabrication

To fabricate the designed metasurface, a bilayer PMMA process is applied for fabrication stability by spin-coating. The resist is baked at 180 ˚C for 3 mins and 5 mins respectively. Moreover, to prevent charging issues during lithography, we introduce an additional conductive layer. Next, we employed the lithography (Elionix ELS-7500) process to define the pattern. After that, we develop the sample by MIBK and IPA in a 1:3 ratio and shake it for 2 minutes and stop the reaction by submerge the sample in IPA for 60 seconds. Subsequently, we deposit a 20 nm chromium layer onto the sample using by thermal evaporator. Afterward, we carried out a lift-off process utilizing remover PG at 90 ˚C for 15 minutes. Then, we utilize the chromium layer as a hard mask to transfer the pattern onto the silicon using an inductively coupled plasma etcher. Finally, the chromium protection layer is removed using Cr-7 (see Figure S15). Finally, the all dielectric metasurface is fabricated and can be characterized by SEM. Imperfections such as edge roughness and dimensional deviations can still introduce additional radiative losses and spectral broadening. To improve sample quality, we propose utilizing high-resolution resists (e.g., ZEP) and incorporating proximity effect correction in electron-beam lithography.

Sample preparation for PL measurement

In preparation for measuring the PbS quantum dots, we subjected the PbS quantum dot solution to a spinning process on the sample at a speed of 1000 rpm for a duration of 60 seconds. This spinning step was performed to ensure better uniformity of the

solution on the sample, followed by a drying step of the sample in air. For the dye case, we apply the cf@870 dye solution onto the sample by spin coating it at 1000 rpm for 60 seconds, ensuring a uniform dispersion. To facilitate drying, we subject the sample to a temperature of 100 °C.

The effect of gap size to Q-factor

The gap size can be defined as follows: gap = $(P - 4R_{ave})/2$, where P is the pitch, and R_{ave} is the average radius in the unit cell.

To evaluate the influence of the gap on the q-BIC behavior, we analysis the data shown in Figure 4. The results show that although the gap affects the overall spectral position and linewidth to some extent, no consistent correlation can be found between gap size and Q-factor. This suggests that the gap-induced near-field coupling is not the dominant factor governing the radiative loss of the q-BIC mode. Instead, our multipole decomposition indicates that the emergence of the quasi-BIC is primarily associated with the excitation of a magnetic quadrupole mode, which originates from the symmetry-broken current distribution inside the resonators. This supports the conclusion that the q-BIC condition is predominantly controlled by symmetry-induced mode interference rather than inter-element spacing alone.

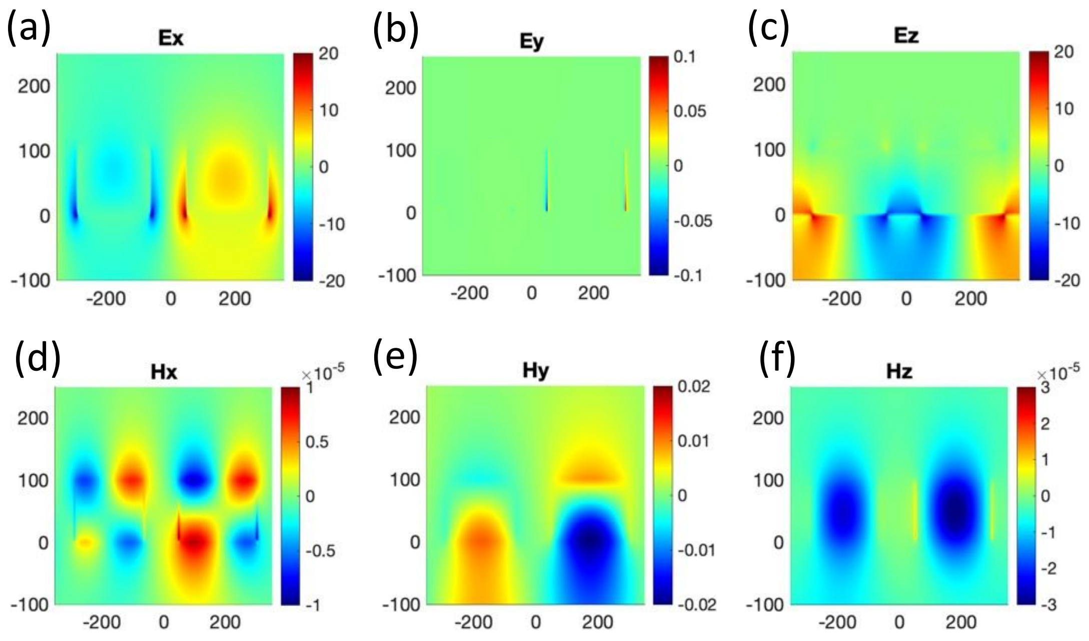

Figure S1. Field profile analysis at the x-z cross-section of the metasurface. (c-e) A resolved electric field corresponds to x, y, and z components. (f-h) A resolved magnetic field corresponds to x, y, and z components. The color bar represents the magnitude of the field.

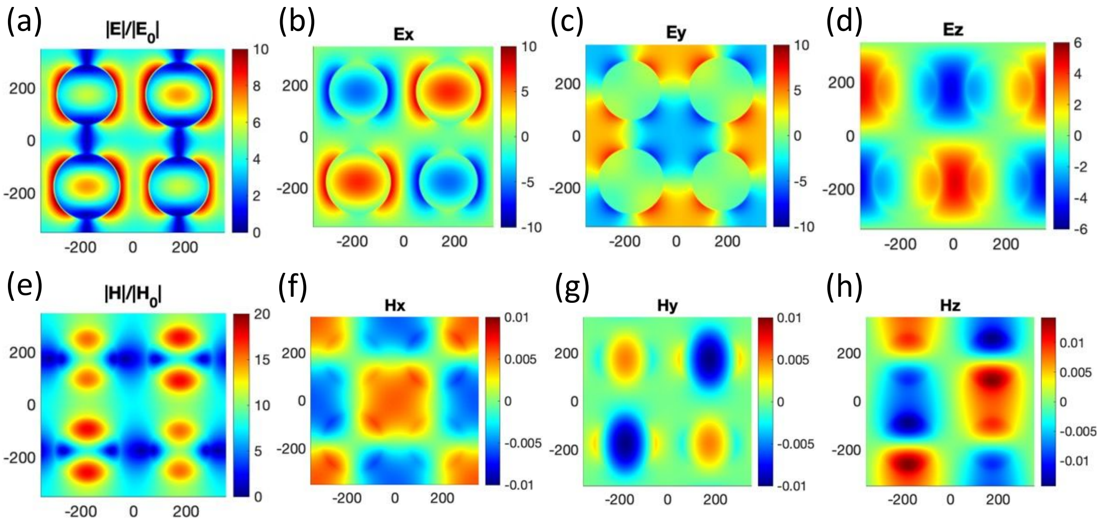

Figure S2. Field profile analysis at the x-y cross-section of the metasurface. (a) Normalized electric field. (b) Normalized magnetic field. (c-e) A resolved magnetic field corresponds to x, y, and z components. (f-h) Resolved magnetic fields correspond to x, y, and z components. The color bar represents the magnitude of the field.

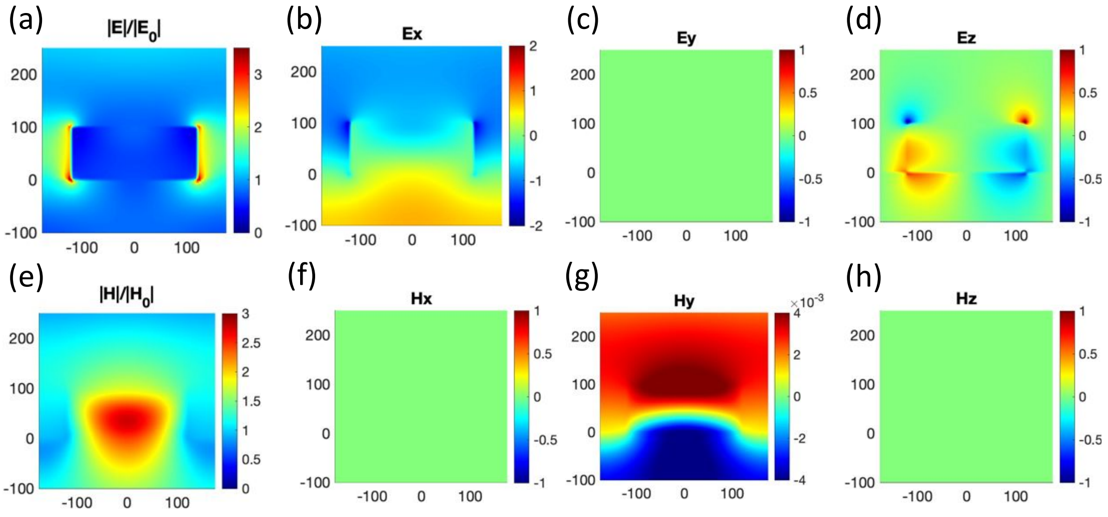

Figure S3. Field profile analysis at the x-z cross-section of the metasurface. (a) Normalized electric field. (b) Normalized magnetic field. (c-e) A resolved magnetic field corresponds to x, y, and z components. (f-h) Resolved magnetic fields correspond to x, y, and z components. The color bar represents the magnitude of the field.

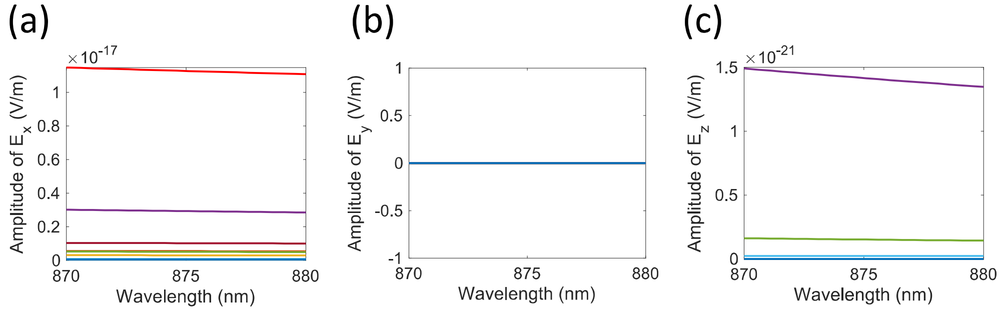

Figure S4. Multipole decomposition of the single radius metasurface. (a) Multipole decomposition of the metasurface of E_x component. (b) Multipole decomposition of the metasurface of E_y component. (c) Multipole decomposition of the metasurface of E_z component.

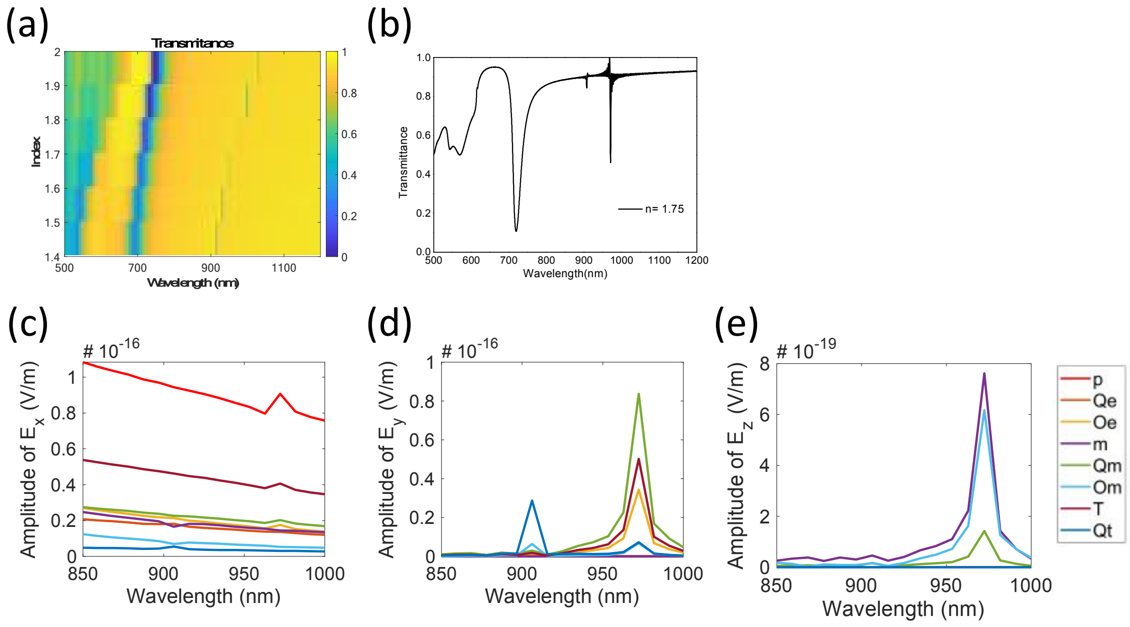

Figure S5. Background index dependence. (a) Transmittance mapping of metasurfaces with varied background index ranges from 1.45 to 1.95. (b) Transmittance spectrum with n = 1.75 (index-matching). Note that the refraction index of sapphire is around 1.75 at the wavelength of interest. (c) Multipole decomposition of E_x component. (d) Multipole decomposition of E_y component. (e) Multipole decomposition of E_z component.

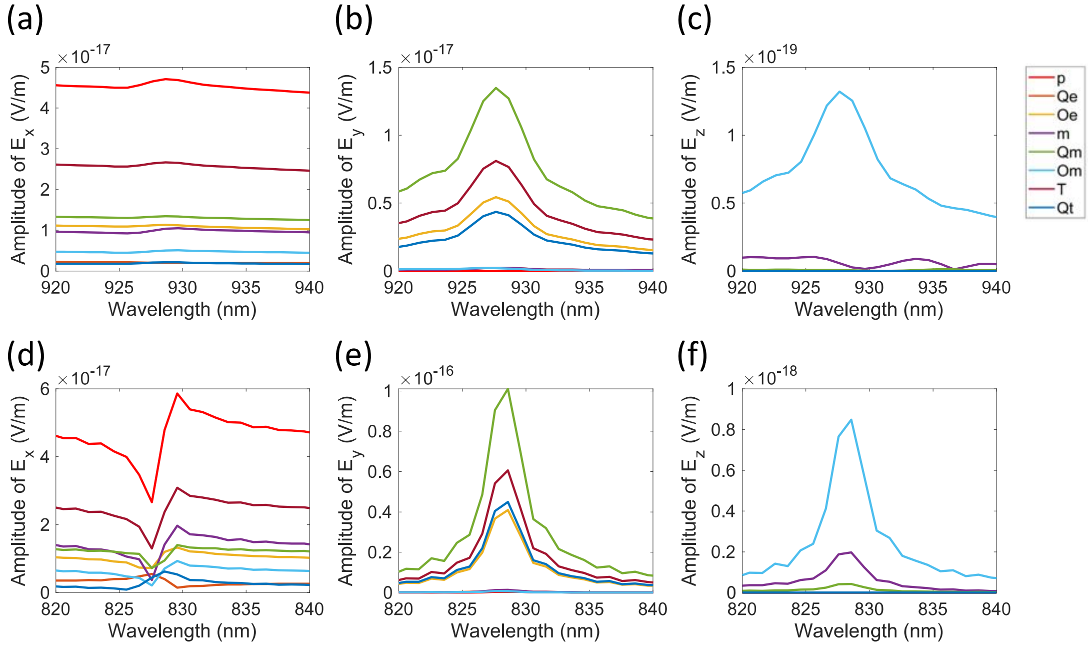

Figure S6. Multipole decomposition analysis with different P. (a-c) E_x, E_y, and E_z components for P= 750 nm. (d-e) E_x, E_y, and E_z components for P= 650 nm.

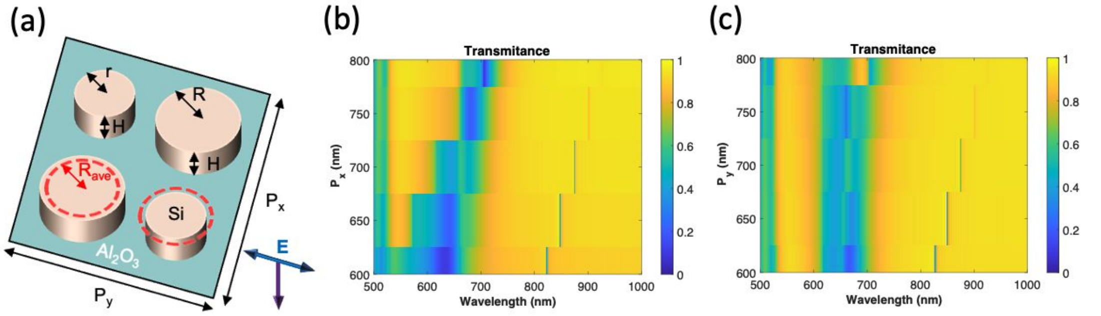

Figure S7. (a) Schematic illustration of the metasurface with bi-radii design. (b) Transmittance mapping of the bi-radii metasurface varied P_x. (c) Transmittance mapping of the bi-radii metasurface with varied P_y. Note that only pitch along one direction is changed.

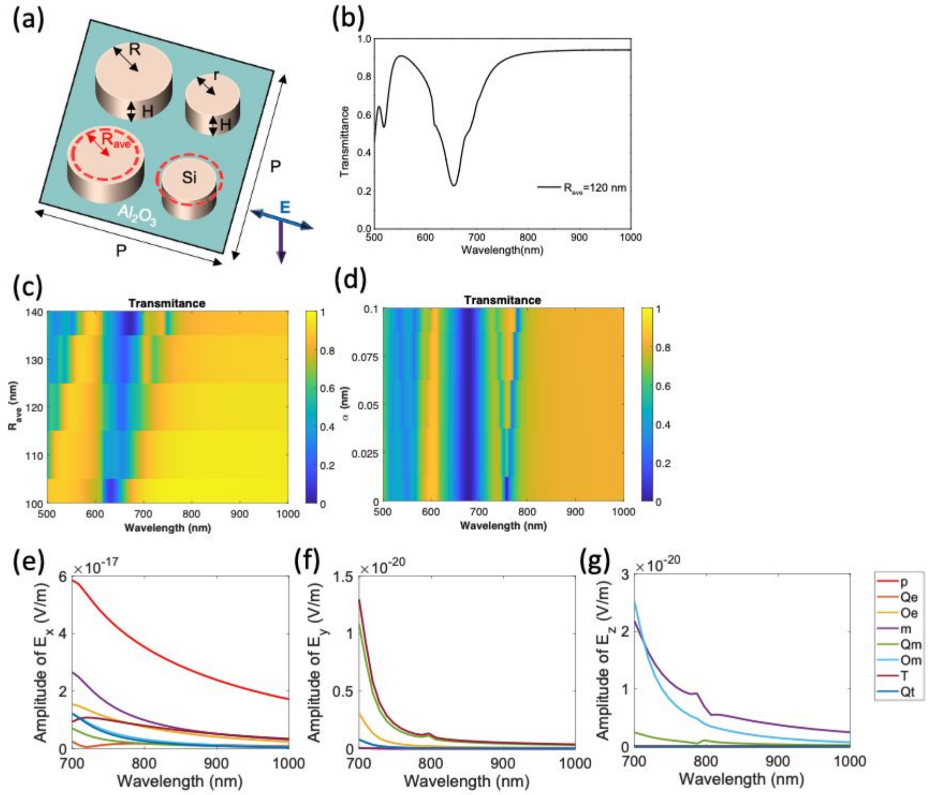

Figure S8. (a) Schematic illustration of the half-bi-radii metasurface unit cell. (b) Transmittance spectrum of the half-bi-radii metasurface of R_{ave}=120 nm. (c) Transmittance mapping of the half-bi-radii metasurface with varied R_{ave}. (d) Transmittance mapping of the half-bi-radii metasurface with varied α. (e-g) Multipole decomposition of the (b) correspond to E_x, E_y, and E_z components.

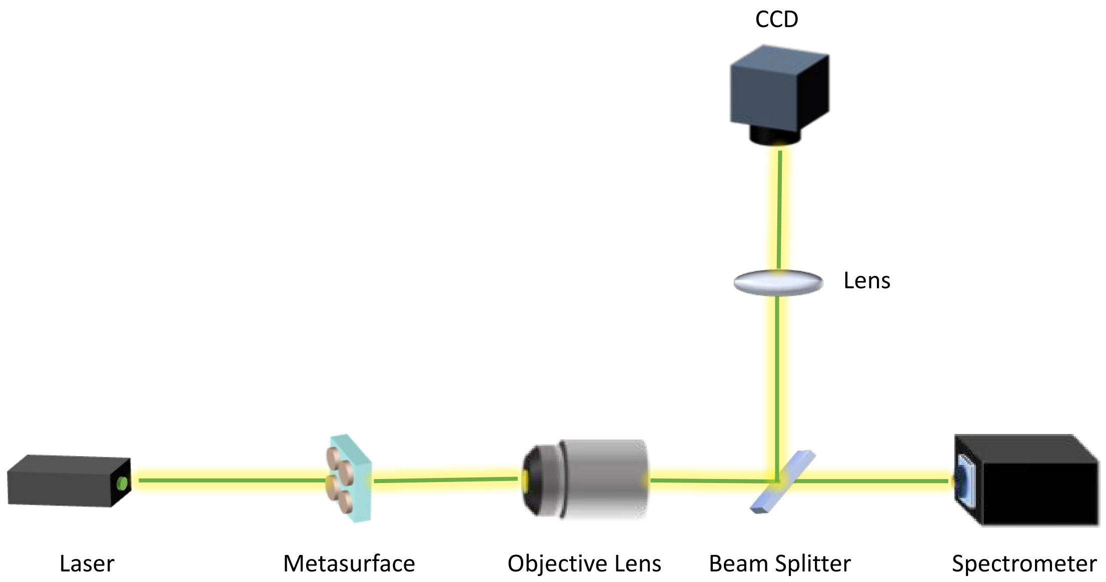

Figure S9. Optical setup for measuring the transmittance spectrum.

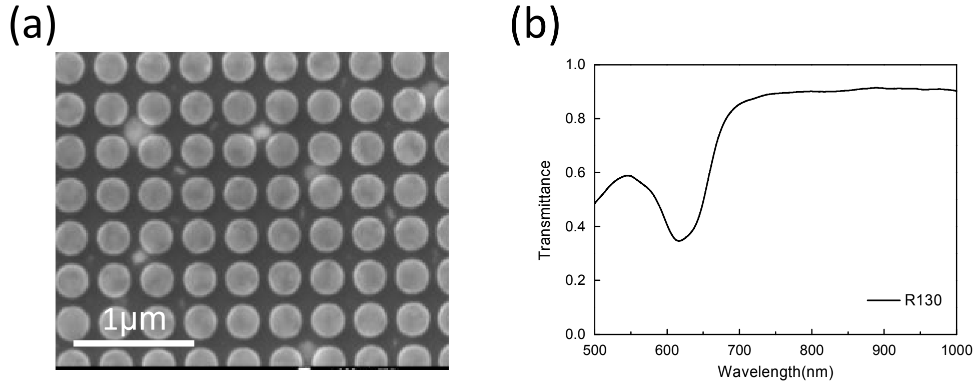

Figure S10. (a)SEM images of the single radius metasurface. (b)Transmittance spectrum correspond to (a)

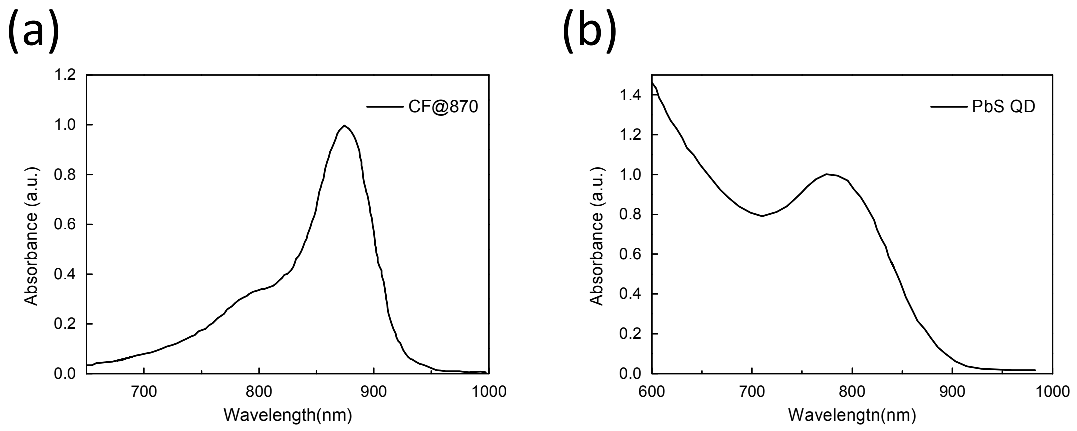

Figure S11. (a)Absorbance spectra of the CF@870 dye. (b)Absorbance spectra of the

PbS quantum dots.

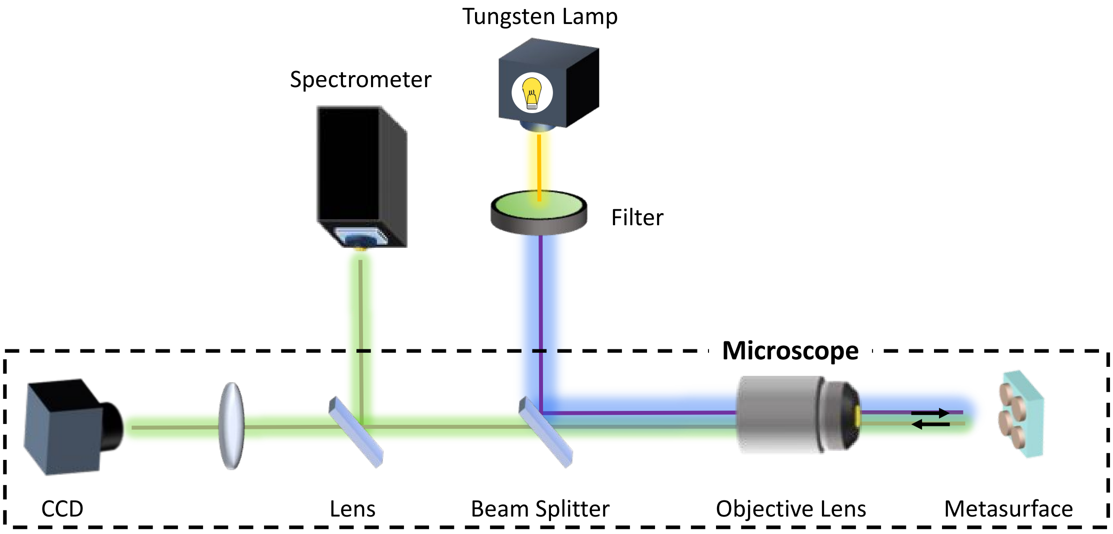

Figure S12. Optical setups for PL of CF@870 dye. The bandpass filter has a center wavelength of 775 nm, enabling light sources between 725 nm and 825 nm to pass through it. The yellow line represents the direct light emission from a tungsten lamp, covering a wide range from 500 nm to 1600 nm. Meanwhile, the purple line represents the light that has been filtered through the bandpass filter. Finally, the green line depicts the photoluminescent (PL) emission, which is the light emitted by the sample after being excited by the filtered light.

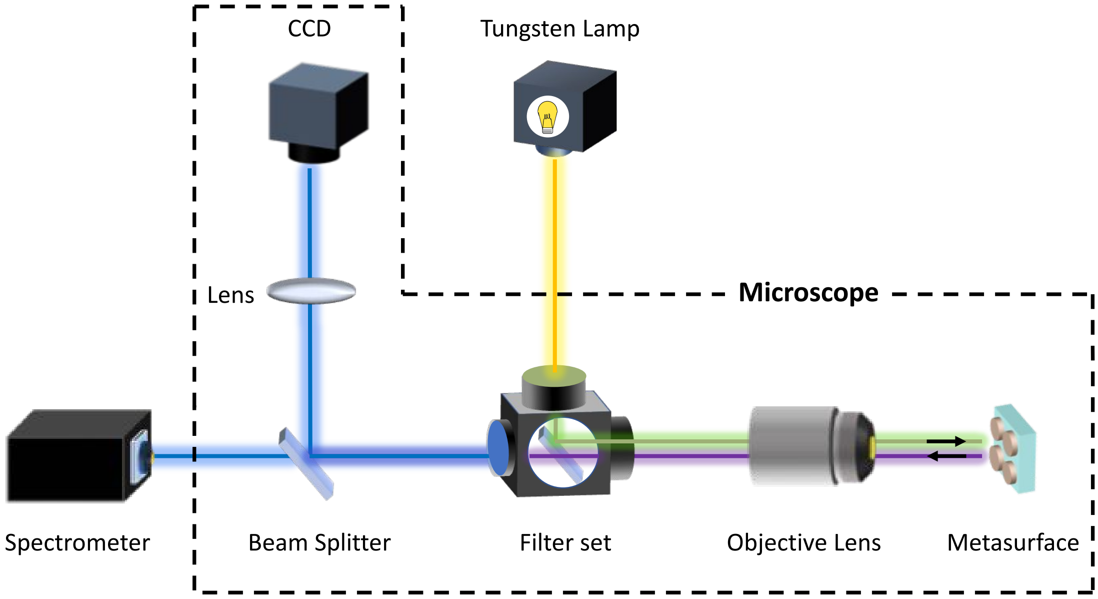

Figure S13. Optical setup for PL of PbS Quantum dots. The yellow line on the graph represents the direct light emission from a tungsten lamp. The bandpass filter with green

color allows the light source between 530 nm to 550 nm to pass through it. Meanwhile, the purple line represents the light that has been emitted from the samples.

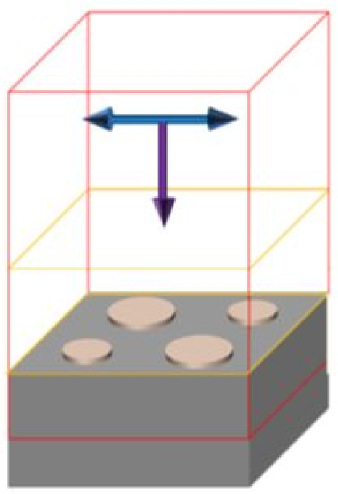

Figure S14. Schematic illustrations of the simulation setup.

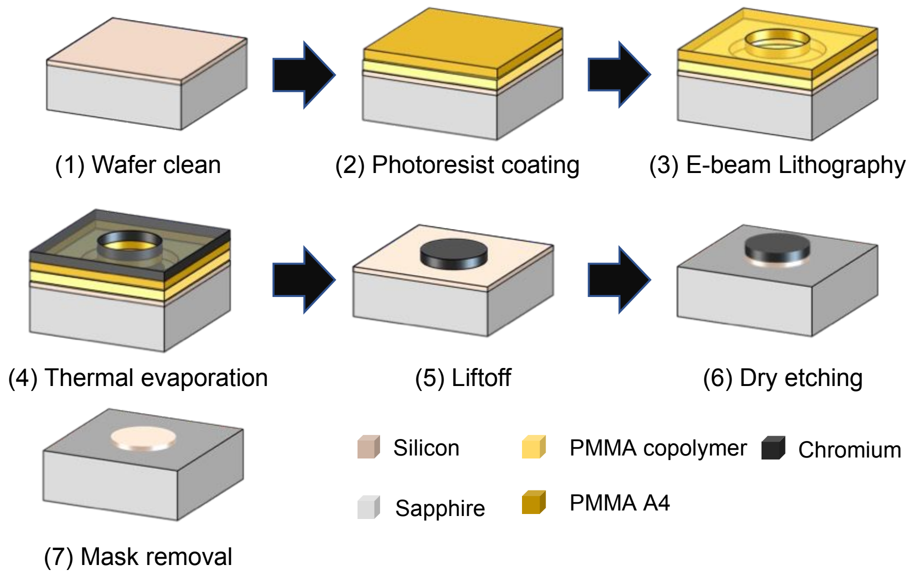

Figure S15. Schematic illustrations of the fabrication process flow.
